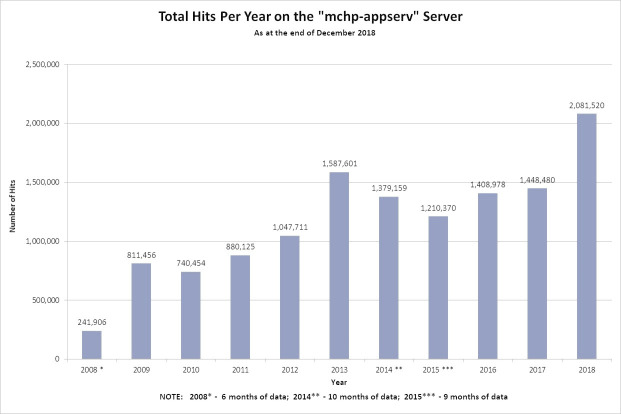# The Concept Dictionary and Glossary at MCHP: Tools and Techniques to Support a Population Research Data Repository

**DOI:** 10.23889/ijpds.v4i1.1124

**Published:** 2019-12-05

**Authors:** M Smith, K Turner, R Bond, T Kawakami, LL Roos

**Affiliations:** 1 Manitoba Centre for Health Policy, Rady Faculty of Health Sciences, University of Manitoba, 408-727 McDermot Ave, Winnipeg, Manitoba, Canada R3E 3P5

## Abstract

The Manitoba Centre for Health Policy’s Concept Dictionary and Glossary, and the Data Repository they document, broaden the analytic possibilities associated with administrative data. The aim of the Repository is to describe and explain patterns of health care and illness, while the Concept Dictionary and Glossary create consistency in documenting research methodologies. The Concept Dictionary alone contains detailed operational definitions and programming code for measures used in MCHP research that are reusable in future projects.

Making these tools available on the internet allows reaching a heterogeneous audience of academic and government health service partners, epidemiologists, planners, programmers, clinicians, and students extending around the globe. They aid in the retention of corporate knowledge, facilitate researcher/analyst communication, and enhance the Centre’s knowledge translation activities. Such documentation has saved countless hours for programmers, analysts and researchers who frequently need to tread paths previously taken by others.

## Introduction

Information-rich environments based on record linkage and cooperation among departments and government ministries have broadened the analytic possibilities associated with administrative data[1]. As a consequence, a number of jurisdictions including Canada, Australia, the United Kingdom, and Scandinavia have proposed or undertaken the development of data repositories[2].

Two key characteristics of these environments – the need to educate prospective researchers concerning content and the importance of issues related to access, privacy and confidentiality – are heightened by the expansion of available data. As topics and requirements change, even experienced analysts need to keep up with newly-installed database holdings and frequently changing privacy requirements. With many studies depending on heterogeneous files from different sources, the need for documentation of working knowledge has never been greater[3].

While introducing the Manitoba Population Research Data Repository (Repository) at the Manitoba Center for Health Policy (MCHP), this paper provides a closer look at one of the many tools the Centre has developed; the Concept Dictionary (CD) and Glossary. These tools aim to aid in the retention of corporate knowledge, facilitate researcher/analyst communication, and enhance the Centre’s knowledge translation activities. We illustrate changes in utilization of these resources over time and note key factors in their development.

## The Manitoba Population Research Data Repository

The Repository is a comprehensive collection of administrative, registry, survey and other databases primarily relating to residents of Manitoba. It was developed to describe and explain patterns of health care and profiles of health and illness, facilitating intersectoral research in areas such as health, education, and social services.

The repository consists of databases grouped into six domains:

- Health―Administrative, survey, and clinical health data (from Manitoba Health, Seniors and Active Living and from Statistics Canada).
- Education ―The Early Development Index (EDI) and Manitoba K–12 data.
- Social ― Housing, Community and Social Services, Healthy Child Manitoba, and survey data.
- Justice―Prosecution files, Court appearances and custodial information.
- Registries―Manitoba Health Insurance, Provider and Metis Registries and Vital Statistics Mortality data.
- Support Files―These files include such things as population counts and the Drug Identification Number (DIN) Master file for working with prescription drug data.

More than 80 different government databases flow into the Repository on an annual basis. All databases are maintained unlinked but are linkable through a common encrypted health identification number after being granted approval from appropriate privacy and access committees.

## The Concept Dictionary and Glossary

Originally many of our information tools were intended only for an internal audience of analysts and programmers. Making them available on the internet (beginning in 1999) meant reaching a larger, more heterogeneous audience of academic and government partners, epidemiologists, planners, programmers, policy makers, clinicians, and students extending around the globe. In part, this was done to help “spread the word” about center research and secondarily to aid in efforts to obtain external funding.

Regardless of the audience, a consistent approach to documentation was needed for describing research methodologies. They can be framed either as best practices or as historical perspectives offering multiple versions. With a best-practice approach, improvements replace previous versions, but require authoritative approval of what is “best”. The historical perspective – currently favoured by MCHP – documents all published approaches to provide the user with choices as well as information on the methodologies used in past research.

While the glossary contains short definitions for commonly used terms, the CD contains detailed operational definitions and programming code for measures used in MCHP research. Concepts are developed where the work is complex and believed to be applicable for future projects. They are written from the original ideas and methods developed for our Deliverables (Reports) and then reviewed and formatted to common standards by the Repository Analyst. As of March, 2019, 364 concepts were available at the MCHP website, along with 2,593 glossary terms. 138 concepts link directly to 218 SAS code files. Of those, 159 are internal (concepts related to the physical location of certain facilities such as the Office of the Public Trustee, methods used to identify First Nations communities, concepts that contain copyrighted material or that would require permission to publish, and SAS code concepts that contain either “intellectual property” or that, based on past experience, require considerable time and resources to support). Pre-defined concept searches provide quick access to measures of health and education and a list of concepts under development (Concepts-in-Progress) is also maintained on the site.

## Applications and Examples

MCHP received Lupina Foundation Funding over the 2005-2010 period to develop web-based documentation in a number of key areas: a) transitioning from the ICD-9-CM to the ICD-10-CA/CCI disease/procedure coding systems, b) costing of the health care system, and c) measuring the social determinants of health and wellbeing. There was insufficient space in this article to describe a fourth area funded by the Lupina Foundation; the development of population health concepts based on pharmaceutical data.

### ICD Environment

Updating our risk-adjustment tools resulted in extensive documentation to assist in the transition that took place in the International Classification of Disease (ICD) environment from ICD-9-CM to ICD-10-CA/CCI. Multiple sources of data were combined to create algorithms based on ICD coding for chronic diseases. A combination of hospital discharge abstracts, medical claims and prescription drug records was used for chronic diseases such as diabetes and hypertension. Comorbidity risk-adjustment tools in the chronic disease population were compared to examine how the Charlson and Elixhauser comorbidity indices can be generalized across jurisdictions. Updating these indices from ICD-9-CM to ICD-10-CA/CCI codes resulted in the large task of incorporating coding changes into the Concept Dictionary[4]. A number of existing MCHP concepts for defining diseases and procedures (and related concepts such as comorbidity indices) had to be redefined and validated.

These indices are very popular tools for assessing comorbidity. The ***Charlson Comorbidity Index*** - both glossary term and concept – has ranked as the most viewed definition in the MCHP Concept Dictionary over many years. Similarly, the ***Elixhauser Comorbidity Index*** concept has periodically emerged within the top 5 most-viewed concepts; while ***Measures of Comorbidity*** has frequently been among the most-viewed concepts.

### Health System Costing

Several MCHP costing reports (for example, Finlayson et al.[5]) updated the methodologies developed to estimate costs for hospital, prescription drug, physician, home care, and long-term care services. These reports were used to update existing MCHP costing concepts and to create a new concept, Costing Methods: An Overview of Costing Health Services in Manitoba

This concept integrates cost data available at MCHP and includes links to related concepts. It includes the most current methods of estimating the costs of health services over time and provides summary information about cost methodologies. This type of information is essential for monitoring longitudinal trends, conducting inter-provincial comparisons, and generating consistent, reliable reports. Such reports have been relied upon by a large audience; since the MCHP report titled *The Additional Cost of Chronic Disease in Manitoba*[5] was first released, it has remained consistently among the top 10 most-downloaded MCHP reports.

### Social Determinants of Health and Wellbeing

Expanding to social research from a focus on health has involved developing a number of population-based measures; some of these do ‘multiple duty’ as indicators of equity/inequity in access, as covariates to help in predicting outcomes, and as variables useful for construction of control groups.

Concepts relating to neighbourhood household income (see Income Quintiles), family structure history, teenage pregnancy, divorce, children in care, employment and income assistance, and residential mobility are also available[6]. Other measures of family characteristics (e.g. family size / number of children), infant health (gestational age, birth weight, Apgar score) and health at several stages in childhood (presence of major health problems, injuries, asthma, mental health diagnoses) have also been created[7]. See the Child Health Indicators concept for more information.

Education data can pose special challenges. Changes in educational policy and testing procedures over time, for example, have increased the difficulty in working with files containing information on test scores, grades, and enrollment. MCHP has created a series of concepts to assist in handling the methodological challenges of dealing with education data. Educational outcomes include several dichotomous variables: late entering school, held back a grade, and old for grade (a combination of the two previous indicators). Several dichotomous variables at the high school level include: high school graduation (within x years of enrolling or by a given age); finished at least Grade 10 (within x years of enrolling or by a given age); and enrollment in a higher-level mathematics course while in high school. Additional indices of educational achievement constructed and validated according to suggestions by Mosteller and Tukey[8] include: the Grade 9 Educational Achievement Index (a combination of grades, credits, and enrollment data); the Grade 12 Language Arts Achievement Index (a combination of test scores and enrollment data), and the Grade 12 Mathematics Achievement Index (a combination of test scores, course data, and enrollment data). Education Overview: Links to Education-Related Data, Concepts and Glossary Terms provides more information about these indicators.

Finally, with the cooperation of the province, data on several new programs have been collected. For example, the Early Development Instrument (EDI), a holistic measure of children’s developmental status at Kindergarten has been administrated in Manitoba every other year since 2005[9]. For more information, see the Early Development Instrument (EDI) Outcomes concept.

## Conclusion

One of several learnings is the value of keeping historical versions of a concept so that changes in data and methodology that impact the concept can be understood and re-created if necessary. This is especially critical for longitudinal studies (some spanning decades) where changes in methods and data are frequently observed. Another was the recognition that the inclusion of information related to the intricacies, nuances and limitations of various data sources has helped to save countless hours of future work repeating the same learning steps. Unfortunately, we have not tried to systematically measure the impact of this on the reduction in work time for future projects, but anecdotally many researchers have commented on it.

Traffic at the MCHP website has increased over time (Figure 1). One of our analytic packages (Deep Log Analyzer) recorded over two million hits in 2018, reflecting the broad interest and appeal of this kind of documentation. Centers like MCHP housing large linkable data repositories accessed by multiple investigators can benefit from web-based resources to compile and disseminate common organizational knowledge. Creating a user-friendly interface to the knowledge generated from a wide range of research projects contributes to productivity and methodological excellence.

**Figure 1:**